# Identification and characterization of novel genetic variants in the first Chinese family of mucopolysaccharidosis IIIC (Sanfilippo C syndrome)

**DOI:** 10.1111/jcmm.18307

**Published:** 2024-04-13

**Authors:** Hongjun Zhao, Lijing Wang, Mengfei Zhang, Huakun Wang, Sizhe Zhang, Junjiao Wu, Yu Tang

**Affiliations:** ^1^ Department of Rheumatology and Immunology, Xiangya Hospital Central South University Changsha China; ^2^ Provincial Clinical Research Center for Rheumatic and Immunologic Diseases, Xiangya Hospital Central South University Changsha China; ^3^ National Clinical Research Center for Geriatric Disorders, Xiangya Hospital Central South University Changsha China; ^4^ Department of Geriatrics, Aging Research Center, Xiangya Hospital Central South University Changsha China; ^5^ Department of Neurology, Xiangya Hospital Central South University Changsha Hunan China

**Keywords:** *HGSNAT*, lysosome, MPS IIIC, mucopolysaccharidosis, transmembrane, variant

## Abstract

Mucopolysaccharidosis type IIIC (MPS IIIC) is one of inherited lysosomal storage disorders, caused by deficiencies in lysosomal hydrolases degrading acidic mucopolysaccharides. The gene responsible for MPS IIIC is *HGSNAT*, which encodes an enzyme that catalyses the acetylation of the terminal glucosamine residues of heparan sulfate. So far, few studies have focused on the genetic landscape of MPS IIIC in China, where IIIA and IIIB were the major subtypes. In this study, we utilized whole‐exome sequencing (WES) to identify novel compound heterozygous variants in the *HGSNAT* gene from a Chinese patient with typical MPS IIIC symptoms: c.743G>A; p.Gly248Glu and c.1030C>T; p.Arg344Cys. We performed in silico analysis and experimental validation, which confirmed the deleterious pathogenic nature of both variants, as evidenced by the loss of HGSNAT activity and failure of lysosomal localization. To the best of our knowledge, the MPS IIIC is first confirmed by clinical, biochemical and molecular genetic findings in China. Our study thus expands the spectrum of MPS IIIC pathogenic variants, which is of importance to dissect the pathogenesis and to carry out clinical diagnosis of MPS IIIC. Moreover, this study helps to depict the natural history of Chinese MPS IIIC populations.

## 1 INTRODUCTION

Mucopolysaccharidosis (MPS) refers to a collection of lysosomal storage disorders (LSD) that arise from deficiencies in lysosomal hydrolases, resulting in the accumulation of acidic mucopolysaccharides (glycosaminoglycans).^1^ Mucopolysaccharides are the main components of connective tissues, including hyaluronic acid, chondroitin sulfate, dermatan sulfate, heparan sulfate and keratin sulfate.^2^, ^3^ These polysaccharides are straight chain heteropolysaccharides that can be combined with a protein peptide chain and polymerize into larger molecules. The degradation of mucopolysaccharides must occur in lysosomes, while over 10 enzymes are known to be involved in their degradation process, with the lack of either enzyme hinders the breakdown of sugar chains. In the LSD patients, excessive mucopolysaccharide accumulates in bone, cartilage and other tissues or organs, thus affecting the normal development of these tissues or organs.^4^

Based on the clinical manifestations and enzyme defects, MPS can be divided into seven major types, and each type is further divided into several subtypes. Of those, MPSIII, also known as Sanfilippo syndrome, is an autosomal recessive metabolic genetic disorder characterized by the accumulation of heparan sulfate in the lysosomes.^5^ Four distinct subtypes of MPS III have been identified based on the genetic deficiencies of specific enzymes: *SGSH* (type A; OMIM 252900), *NAGLU* (type B; OMIM 252920), *HGSNAT* (type C; OMIM 252930) and *GNS* (type D; OMIM 252940).^6^ Among them, MPS IIIC (Sanfilippo C) is a multi‐system MPS that begins in early childhood and is marked by progressive degeneration of the central nervous system, severe mental retardation and other neurological symptoms.^7^, ^8^, ^9^ Other clinical symptoms include skeletal and muscular issues (such as joint stiffness, scoliosis, hip dysplasia and contractures), hearing loss, respiratory and sinus infections, and heart problems.^8^, ^9^ Specifically, MPS IIIC is caused by mutations in the *HGSNAT* gene, which encodes acetyl‐CoA:α‐glucosaminide N‐acetyltransferase (EC 2.3.1.78), a lysosomal transmembrane enzyme. This enzyme catalyses the acetylation of the terminal glucosamine residues of heparan sulfate before it is hydrolyzed by α‐N‐acetyl glucosaminidase (NAGase).^10^ The *HGSNAT* gene was identified as being responsible for MPS IIIC in 2006,^11^, ^12^ and since then, around 72 *HGSNAT* mutations have been reported (from HGMD).

MPS is a rare disease, accounting for less than 0.1% of all genetic diseases. The prevalence of different types of MPS is related to race and geography,^13^ with MPS type II mainly found in Asian population, and MPSI and MPSIII types in Europe. In particular, MPS IIIC and D were less prevalent in most populations, with estimates of MPS III prevalence ranging from 1 to 9 per 1 million individuals.^14^ So far, few studies have focused on the genetic landscape of MPS IIIC in China, where IIIA and IIIB were the major subtypes.^15^, ^16^, ^17^

In this study, we identified compound heterozygous *HGSNAT* variants in a Chinese patient with MPS IIIC: c.743G>A; p.Gly248Glu and c.1030C>T; p.Arg344Cys. Through in silico analysis and experimental validation, we confirmed that these variants are pathogenic and result in complete loss of HGSNAT enzyme function and failure of lysosomal localization. To the best of our knowledge, the MPS IIIC is first confirmed by clinical, biochemical and molecular genetic findings in China. Our study thus expands the mutation spectrum of MPS IIIC pathogenic genes, which is of importance to clarify the pathogenesis of MPS IIIC and to carry out genetic diagnosis.

## 2 MATERIALS AND METHODS

### 2.1 Subjects

A Chinese family, in which the proband had MPS IIIC, along with all available family members, were recruited at Xiangya Hospital of Central South University, Hunan, China. Blood samples were obtained from all family members using vacutainer tubes containing EDTA. The study was approved by the Institutional Review Board of Xiangya Hospital of Central South University (#2022020132) and adheres to the principles of the Declaration of Helsinki. Written informed consent was provided by all participants.

### 2.2 WES analysis

The phenol‐chloroform extraction method was used to isolate genomic DNA from peripheral blood of the proband.^18^ Yikon Medical Laboratory Co., Ltd. (Shanghai, China) conducted WES on the proband (II:6) using the HiSeq 2000 platform (Illumina) and the SureSelect Human All Exon V6 kit (Agilent) for exome capture, according to the manufacturer's manual. The exome library was constructed using 350 ng genomic DNA that was sheared into 150–200 bp for enrichment using the Covaris instrument (Covaris). The platform collected 101 bp pair‐end reads for sequencing the enrichment libraries for target regions. The genomic regions harbouring candidate variants were further amplified by PCR and Sanger sequencing in all family members.

### 2.3 In silico analysis

The variants were evaluated and annotated with a list of servers and databases including SIFT, Polyphen‐2, MutationTaster, CADD, gnomAD, dbSNP, ClinVar, ChinaMAP and HUABIAO. The pathogenicity was further predicted with the algorithms, most recently empowered by machine learning, deep learning or neural network model, such as EVE,^19^ gMVP (version 2021‐02‐28),^20^ PrimateAI (v0.2),^21^ AlphaMissense,^22^ MutFormer^23^ and MAVERICK.^24^ The predictions were also carried out with InterVar (version 2022‐06‐13),^25^ based on the ACMG/AMP 2015 guidelines. The topologies of transmembrane HGSNAT were described by DeepTMHMM (version 1.0.15)^26^ and TOPCONS 2.0,^27^ involving several other topology‐prediction tools. The conservation of variant sites was manifested by WebLogo3.^28^ The structure of HGSNAT protein was predicted by AlphaFold,^29^ and visualized and edited with PyMOL (Schrodinger, version 2.5.4). The stability of proteins were assessed by I‐Mutant 2.0.^30^

### 2.4 Overexpression plasmids

To construct overexpression plasmids, the vector pUC57‐HGSNAT::MYC::FLAG was synthesized (Tsingke Bio., Beijing, China). The fragment of HGSNAT::MYC::FLAG was amplified by PCR and ligated into the pCSC vector^31^ with AgeI/BsrGI double digestion. The obtained vector served as the backbone for site‐directed mutagenesis to construct expression plasmids with genetic variants: c.743G>A; p.Gly248Glu, c.1030C>T; p.Arg344Cys and c.710C>A; p.Pro237Gln, respectively. All constructed plasmids were confirmed by Sanger sequencing before use.

### 2.5 Cell culture

293T cells (Procell) were routinely cultured in a humidified incubator at 37°C with 5% CO_2_. Dulbecco's Modified Eagle Media (DMEM) supplemented with 10% fetal bovine serum (FBS) and 1% each of penicillin, streptomycin and amphotericin B (Beyotime) was used as the cell culture medium.

### 2.6 Cellular HGSNAT/NAGase activity

293T cells were seeded into a 6‐well plate and transfected, on the next day, with 1 μg of plasmids (pCSC‐HGSNAT::MYC::FLAG) by polyethylenimine (PEI) respectively. Forty‐eight hours later, cells were washed with PBS for three times. Cells were then lysed with H_2_O and collected into EP tubes for sonication. The concentration of obtained proteins were measured by the BCA method with a protein assay kit (Pierce), according to the manufacturer's protocol.

In a 96‐well plate, add 10 μL protein homogenate, 5 μL McIlvain buffer (pH 5.5), 5 μL 3 mM substrate (MU‐βGlcNH_2_, Biosynth, #EM31025) or 3 mM substrate (MU‐βGlcNAc, Biosynth, #M5504) and 5 μL 5 mM Acetyl‐CoA (Sigma, #A2056). Incubate at 37°C for 1 h and add 225 μL 0.4 M Glycine buffer (pH 10.4) to quench the reaction. The mixture was immediately monitored at 360 nm excitation and 450 nm emission wavelengths with a multi‐mode plate reader (Cytation 5; BioTek). The HGSNAT / NAGase specific activity was calculated as:

Fluorescence × 0.25 × 1 × 1 = nmol/h/mg.

Standard curve slope 0.01 1 h homogenate (mg/mL).

### 2.7 Western blot and immunostaining

For western blot, 293 T cells were seeded in a 6‐well plate and the next day transfected with 1 μg of plasmids respectively with PEI (pCSC‐HGSNAT::MYC::FLAG). Twelve hours later, the cells were harvested with 200 μL of RIPA buffer (Beyotime). Then, each sample was added with 4× loading buffer and 2‐ME and heated for 8 min at 55°C. Twenty micrograms of each protein was then separated using 10% SDS‐PAGE and then transferred to a PVDF membrane (Millipore). The blots were blocked with 5% skimmed milk, and later incubated with diluted primary antibodies, including MYC (Proteintech #60003‐2‐Ig, 1:2000) and GAPDH (Abclonal #AC033, 1:20,000), overnight at 4°C. The next day, the blots were washed three times with TBST at a 15‐min interval, and later incubated with the diluted secondary antibodies conjugated with HRP for 1 h at room temperature. After washing with TBST, western blots were detected using the ECL substrate and eventually visualized using the ChemiDoc XRS imaging apparatus (Bio‐Rad).

For immunostaining, 293 T cells were seed on coverslips of a 24‐well plate, and transfected with 250 ng plasmids (pCSC‐HGSNAT::MYC::FLAG) and the reporter plasmid LAMP1::mCherry, respectively. After 48 h, cells were washed with PBS and fixed with 4% PFA. Cells were then incubated with BSA blocking buffer and further diluted primary antibody: FLAG (Abclonal #AE005, 1:100), at 4°C for overnight. Cells were washed with PBST and incubated with secondary antibody conjugated with AlexaFluor488 (Invitrogen #A21202, 1:500) at room temperature for 1 h. Finally, cells were counter‐stained with DAPI and mounted with anti‐fade PVA for further observation under confocal microscopy.

### 2.8 Statistics analysis

GraphPad Prism software (version 8.0.1) was utilized for statistical analysis and graphing. The Student's *t*‐test was used for statistical analysis, with a 95% confidence level considered the significance of differences between groups. A *p* < 0.05 indicates statistically significant.

## 3 RESULTS

### 3.1 Clinical description and molecular analysis

**FIGURE 1:**
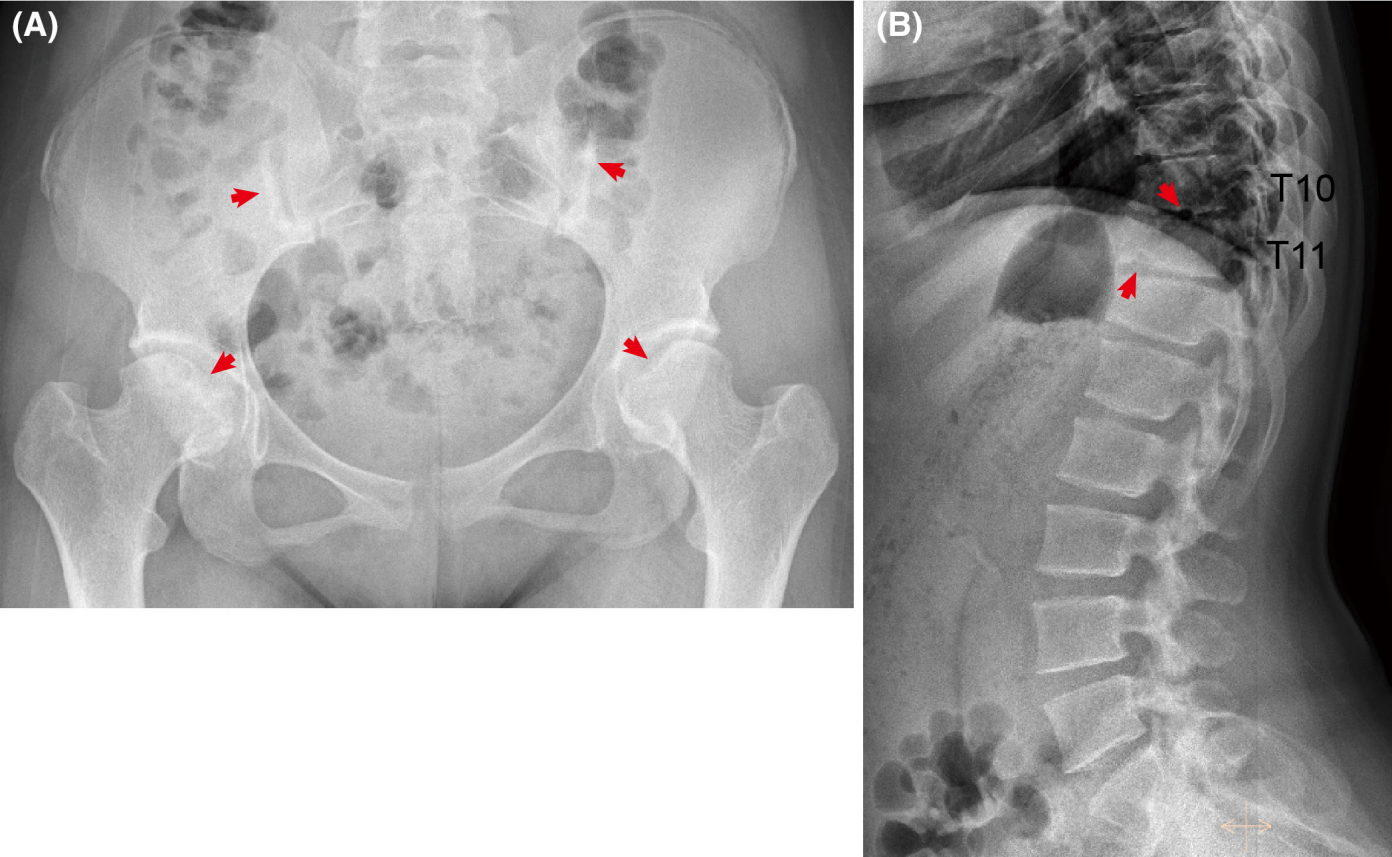
Clinical manifestations of the proband. (A) The x‐ray examination showed that the bilateral femoral head became flattened; multiple cystic low‐density changes under the bilateral hip surface (red arrows), as well as increased bone density of the bilateral sacroiliac joint surface (red arrows). (B) The vertebral sequence was slightly discontinuous; the lower margin of T10 and T11 vertebrae showed the formation of Schmorl's nodes (red arrows), and the corresponding vertebral body was slightly flattened.

**FIGURE 2:**
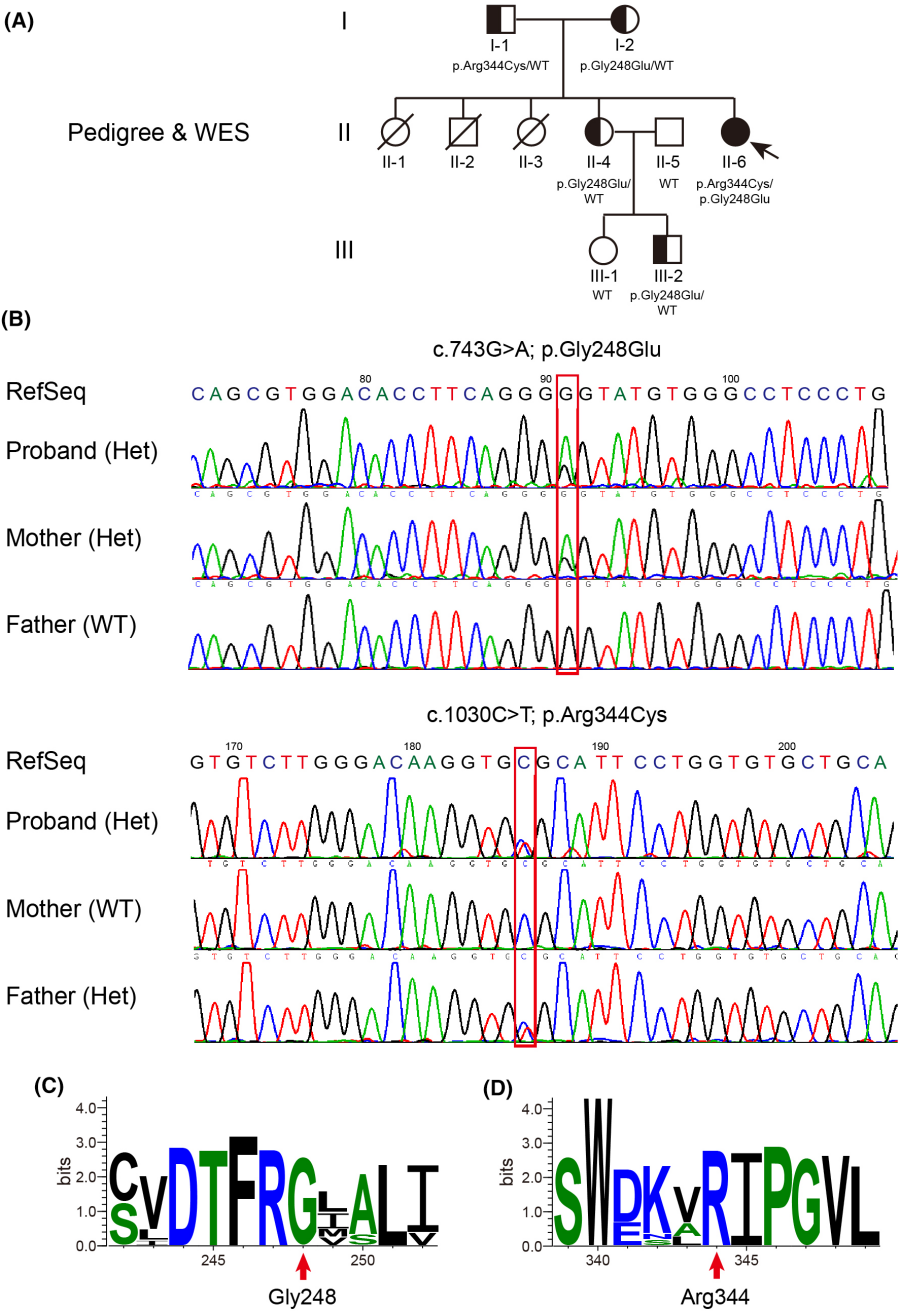
The family pedigree and molecular analysis. (A) The family pedigree shows the proband (II6, arrow) in an autosomal recessive pattern over three generations. (B) Identification and co‐segregation of compound heterozygous *HGSNAT* variants by WES analysis and Sanger sequencing verification. The variant, c.1030C>T; p.Arg344Cys, is inherited from the father and a novel variant, c.743G>A; p.Gly248Glu, is derived from the mother. (C, D) The evolutionary conservation of the variant positions among HGSNAT homologues across vertebrates.

In our study, the proband, a 15‐year‐old Chinese girl, was admitted for clinically performing both‐hip pain and mental retardation examinations. Specifically, the proband experienced pain and discomfort in both hips 5 years earlier and was unable to walk in severe cases. The x‐ray examination showed that the bilateral femoral head became flattened; multiple cystic low‐density changes under the bilateral hip surface, as well as increased bone density of the bilateral sacroiliac joint surface (Figure 1A). Still with the physiological curvature of the spine, the vertebral sequence was slightly discontinuous; the lower margin of T10 and T11 vertebrae showed the formation of Schmorl's nodes, and the corresponding vertebral body was slightly flattened (Figure 1B). In addition, the proband is currently in grade 6 of primary school, with poor grades and cannot read by herself, suggesting that she has serious intellectual defects (data not provided). Her parents were all phenotypic normal and had non‐consanguineous marriages. She has an older sister, who is normal and bearing two normal children, as well as three siblings who were already dead (Figure 2A).

To investigate the possibility of an inherited cause, we collected a peripheral venous blood sample from the proband for whole‐exome sequencing (WES). The analysis revealed novel compound heterozygous variants in the *HGSNAT* gene: c.1030C>T (chr8:43037305 (hg19); NM_152419.3) and c.743G>A (chr8:43025837 (hg19); NM_152419.3). We subsequently performed targeted Sanger sequencing of the *HGSNAT* gene in other family members, revealing either a heterozygous variant or unaffected health status (Figure 2A,B). Considering this, the proband was thus diagnosed as MPS IIIC, which is rarely seen within the Chinese population.

Specifically, we confirmed that one variant (c.1030C>T), inherited from the father, leads to protein change (p.Arg344Cys); whereas the other variant (c.743G>A), inherited from the mother, causes protein change (p.Gly248Glu) that is novel and unappreciated before, after a thorough search of public databases and Chinese cohorts, including gnomAD, dbSNP, ClinVar, ChinaMAP and HUABIAO, among others (Figure 2B; Table 1).

**TABLE 1:** In silico prediction of the potential effects of HGSNAT variants.

Amino acids	SIFT		Polyphen2		MutationTaster		CADD		gnomAD Exome	dbSNP	ClinVar
	Score	Prediction	Score	Prediction	Score	Prediction	Score	Prediction			
p.Gly248Glu	0	Damaging	1	Probably damaging	1	Disease causing	25.4	Damaging	‐	‐	‐
p.Arg344Cys	0.003	Damaging	1	Probably damaging	1	Disease causing automatic	34	Damaging	1.218e‐05	rs121908285	Accession: 1237 Phenotype: MPS IIIC Clinical significance: Pathogenic
p.Pro237Gln	0.223	Tolerable	0.151	Benign	1	Polymorphism	8.451	Tolerable	2.992e‐05	rs727503962	Accession: 167177 Phenotype: not specified Clinical significance: Benign

### 3.2 In silico analysis of variants

The *HGSNAT* variants result in the substitution of proteins at amino acid position 344 and 248 respectively. Those positions are highly conserved in HGSNAT homologues across vertebrates after the evolutionary conservation analysis by WebLogo (Figure 2C,D). Functional prediction of both variants was conducted by a list of bioinformatics algorithms, including SIFT, Polyphen‐2, MutationTaster, CADD and others, favoured a deleterious effect (Table 1). We further predicted the pathogenicity with the most recent algorithms, empowered by machine learning, deep learning or neural network model, such as EVE, gMVP, PrimateAI, AlphaMissense, MutFormer and MAVERICK, the p.Gly248Glu/p.Arg344Cys variants were all scored as highly pathogenic (Figure S1). As a control, we also examined another reported *HGSNAT* variant (p.Pro237Gln), which is benign and caused by a single nucleotide polymorphism (SNP) known as rs727503962. Moreover, to standardize the clinical interpretation of genetic variants according to the ACMG/AMP 2015 guidelines, we carried out the prediction with the help of InterVar (https://wintervar.wglab.org/). The p.Gly248Glu variant is of uncertain significance (PM2‐moderate, PM5‐moderate and PP3‐supporting), while the p.Arg344Cys variant is likely pathogenic (PM1‐moderate, PM2‐moderate, PP3‐supporting and PP5‐supporting) (Table 2).

**TABLE 2:** Pathogenicity predictions based on the ACMG/AMP 2015 guidelines.

	p.Gly248Glu	p.Arg344Cys
InterVar	Uncertain significance	Likely pathogenic
PVS1	0	0
PS1	0	0
PS1 grade	1	1
PS2	0	0
PS2 grade	1	1
PS3	0	0
PS3 grade	1	1
PS4	0	0
PS4 grade	1	1
PS5	0	0
PS5 grade	1	1
PM1	0	1
PM1 grade	2	2
PM2	1	1
PM2 grade	2	2
PM3	0	0
PM3 grade	2	2
PM4	0	0
PM4 grade	2	2
PM5	1	0
PM5 grade	2	2
PM6	0	0
PM6 grade	2	2
PM7	0	0
PM7 grade	2	2
PP1	0	0
PP1 grade	3	3
PP2	0	0
PP2 grade	3	3
PP3	1	1
PP3 grade	3	3
PP4	0	0
PP4 grade	3	3
PP5	0	1
PP5 grade	3	3
PP6	0	0
PP6 grade	3	3

### 3.3 Diminished HGSNAT activities caused by the variants

**FIGURE 3:**
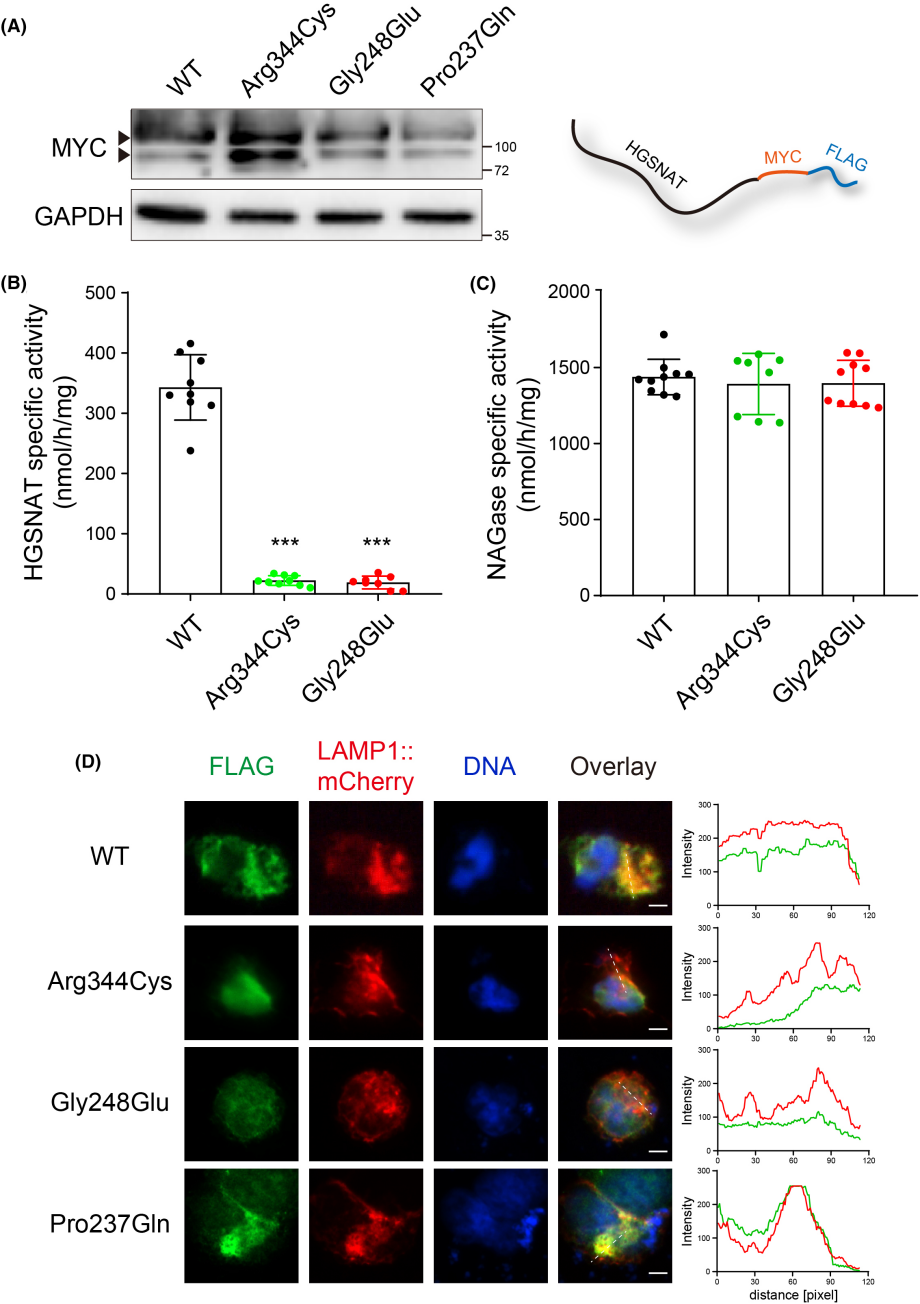
Diminished HGSNAT activities and failed lysosomal localization caused by the variants. (A) Expression vectors containing the full‐length of WT and mutant HGSNATs were respectively transfected in 293 T cells. Detection of overexpressed HGSNAT::MYC::FLAG by western blot within a short time of 12 h. (B, C) The specific activities of HGSNAT and NAGase were respectively assessed at 48 h after transfection. ***p < 0.001. (D) To examine if the mutant HGSNAT proteins were sorted to its target location, the lysosome, 293 T cells were co‐transfected with both HGSNAT expression vectors and LAMP1::mCherry, a lysosome reporter, for 48 h and fixed for immunostaining. Analysis of cells by confocal microscope revealed that LAMP1::mCherry co‐localized with HGSNAT^WT^ and HGSNAT^Pro237Gln^ proteins, whereas HGSNAT^Arg344Cys^ or HGSNAT^Gly248Glu^ exhibited limited overlapping with lysosomes. Scale bars, 10 μm.

To further evaluate the potential deleterious impacts of the variants on HGSNAT, we generated plasmids expressing the full‐length wild‐type (WT) and mutant HGSNAT (p.Arg344Cys and p.Gly248Glu), respectively. To facilitate further biochemical assays, the HGSNAT ORF was C‐terminally fused with two small tags, MYC (~1.2 kDa) and FLAG (~1 kDa) (Figure 3A). Equal amounts of these plasmids were then transiently transfected into 293 T cells, which produced HGSNAT::MYC::FLAG proteins within a short time of 12 h (Figure 3A). Forty‐eight hours later, HGSNAT activity was found to be significantly increased in the HGSNAT^WT^ transfected cells (average 342.903 nmol/h/mg), whereas both mutant HGSNATs resulted in diminished HGSNAT activities (average 22.2692 and 19.0039 nmol/h/mg, respectively) (Figure 3B). As a control, the NAGase specific activities were comparable among all groups (Figure 3C), reminiscent of the type IIIC specificity.

To examine if the mutant HGSNAT proteins were correctly localized to lysosomes, 293 T cells were co‐transfected with both HGSNAT constructs and LAMP1::mCherry, a lysosome reporter, for 48 h and fixed for immunostaining. Examination of cells via confocal microscope demonstrated that LAMP1::mCherry co‐localized with HGSNAT^WT^ and HGSNAT^Pro237Gln^ proteins, whereas HGSNAT^Arg344Cys^ or HGSNAT^Gly248Glu^ exhibited limited overlapping with lysosomes (Figure 3D). The diminished enzyme activities, as well as the failure in lysosomal location, thus confirmed the pathogenic effects of the variants.

### 3.4 The underpinnings of detrimental effects caused by the variants

**FIGURE 4:**
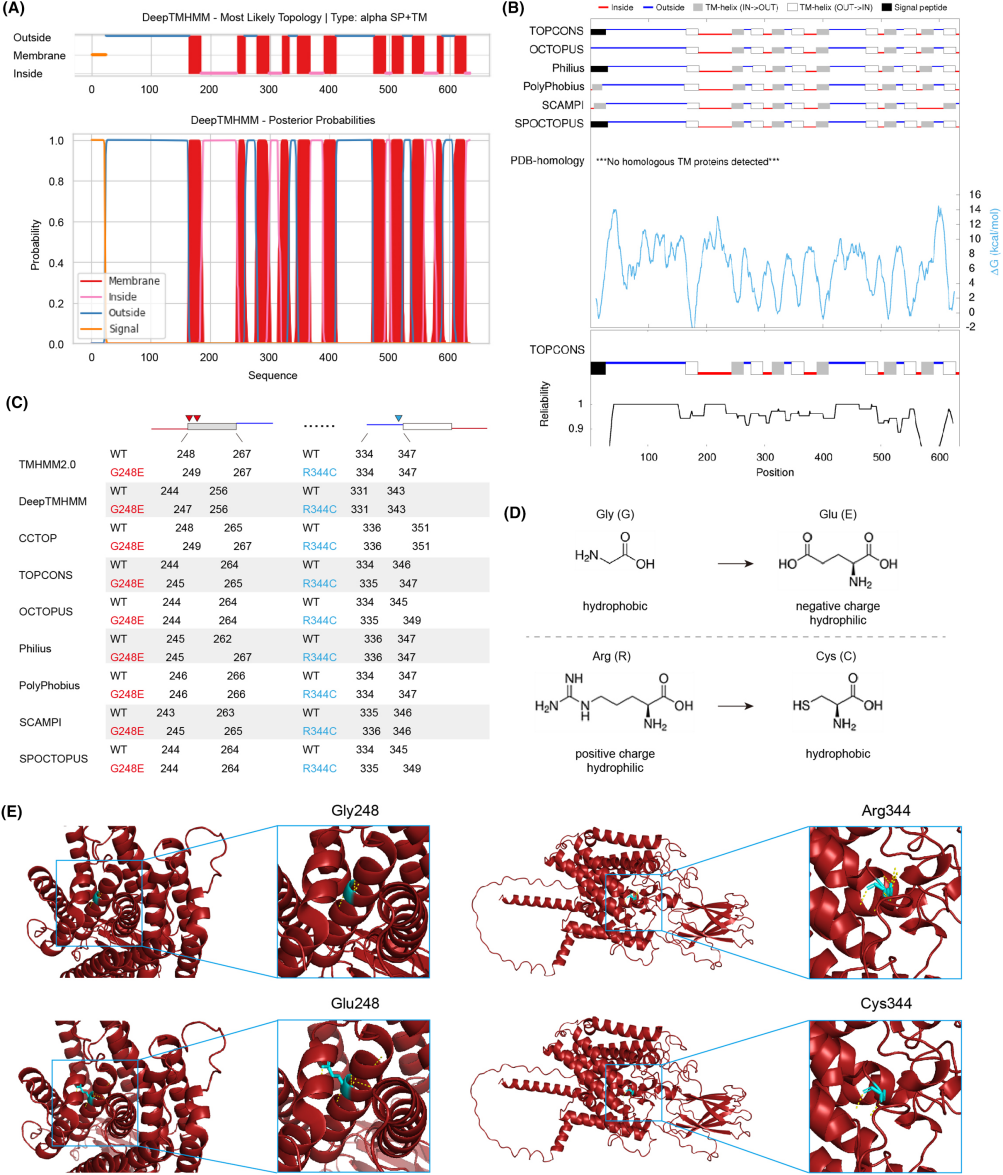
In silico analysis of the membrane topology and detrimental effects caused by the variants. (A, B) In silico analysis by DeepTMHMM and TOPCONS predicted the topologies of HGSNAT enzyme with 11 transmembrane helices in the lysosomal membrane. (C) The p.Gly248 site was predicted to be positioned either on the boundary of or inside the lumen area of a transmembrane segment, while the p.Arg344 position lies in the domain exposed into the lysosome. (D) The p.Gly248Glu variant alters the hydrophobic Gly into hydrophilic negative charged Glu, it is thus highly possible that the variant contributes to the unstable topology of the transmembrane segment. Likewise, the p.Arg344Cys variant changes the hydrophilic positive charged Arg into hydrophobic Cys, the surrounding chemical bonds are likely to be remould. (E) The protein structure of HGSNAT was predicted by AlphaFold, and potential alterations of hydrogen bonds (yellow dashed lines) caused by mutations were visualized and edited with PyMOL.

As human HGSNAT is a transmembrane protein that undergoes glycosylation, there is currently no crystal structure available for it, which poses additional difficulties in assessing the functional impact of its variants. In silico analysis by DeepTMHMM and TOPCONS predicted the topologies of HGSNAT enzyme with 11 transmembrane helices in the lysosomal membrane (Figure 4A,B). As predicted by a list of web servers, the p.Gly248 position was placed either on the edge of or inside the lumen area of a transmembrane segment, while the p.Arg344 position was located in the domain exposed into the lysosome (Figure 4C). The p.Gly248 variant appears not to dampen the formation of transmembrane helices as a whole (Figure 4C), however, considering that the p.Gly248Glu variant alters the hydrophobic Gly into hydrophilic negative charged Glu, it is highly possible that the variant contributes to the unstable topology of the transmembrane segment (Figure 4D). Likewise, the p.Arg344Cys variant changes the hydrophilic positive charged Arg into hydrophobic Cys, the surrounding chemical bonds are likely to be remould (Figure 4D). This might be further reinforced by appreciating the protein structure predicted by AlphaFold, which showed essential alterations of hydrogen bonds caused by the variants (Figure 4E). Additionally, we also evaluated the effect of protein mutations of HGSNAT (UniProt Q68CP4) by I‐Mutant, and showed that both variants may lead to large decrease of stability (Table S1).

## 4 DISCUSSION

This study characterizes compound heterozygous *HGSNAT* variants that were identified in a Chinese patient exhibiting typical symptoms of MPS IIIC: c.743G>A; p.Gly248Glu and c.1030C>T; p.Arg344Cys. We verified them both as detrimental pathogenic variants and dissected the possible underpinnings, by both in silico analysis and experimental validation. The MPS IIIC is, by far, first confirmed in China, which may help to outline the natural history of Chinese MPS IIIC populations. Note that a recent study has reported one case of MPS IIIC out of 34 Chinese patients with MPS III, to be frustrated, no reasonable/clear variants were found out in that case.^15^ The majority of MPS cases in China remains I, II, IIIA and IIIB.^15^, ^16^, ^17^

Revisiting from another in silico perspective, as the variant c.743G>A just located on the boundary between exon 7 and 8, it is possible that the variant may interfere with the RNA splicing process. However, since the coding mutant of p.Gly248Glu has been proved to diminish the HGSNAT activity, the splicing possibility becomes greatly dwarfed. Alternatively, its effect on the transmembrane helix merits further experimental tests. To this end, several assays might be feasible.

The first assay involved the *Escherichia. coli* inner membrane protein leader peptidase, which can detect the precise free energy (ΔGapp) of translocon‐mediated integration of transmembrane helices into the membrane. This assay quantified the proper integration of the transmembrane segment, including both WT and mutants.^32^, ^33^ Another approach to investigate the topology of HGSNAT involves a Cys‐accessibility assay, wherein the reagent 4‐acetamido‐4′‐maleimidylstilbene‐2,2′‐disulfonic acid (AMS), which cannot permeate the membrane, is used to react with Cys residues; Only Cys residues that are exposed to the cytosol and not shielded by the membrane will undergo this reaction.^34^, ^35^ The assay successfully validated the possible topology changes by CFTR p.Gly91Arg and p.Gly85Glu variants, and specifically, p.Gly85Glu misfolding is based in transmembrane destabilization by Glu and loss of Gly.^35^ Interestingly, the membrane topology can also be assessed by biophysical avenues, for example, atomistic simulations and nuclear magnetic resonance (NMR) measurements. Through the use of those techniques, Xu et al. illustrated that a single‐residue polymorphism in the transmembrane domain of FcγRIIB, known as FcγRIIB‐T232, which is linked to systemic lupus erythematosus (SLE) across global populations, resulted in a significant increase in the bending angle of the transmembrane helix. This led to a more tilted orientation in the lipid membrane, thereby decreasing the lateral mobility and inhibitory capabilities of FcγRIIB.^36^, ^37^

While this study expands the spectrum of MPS IIIC pathogenic variants and facilitates clinical diagnosis, gene editing efforts to restore the HGSNAT activity have also been devoted. The multi‐pass transmembrane biology of HGSNAT leads to its own challenges, compared to those soluble secreted proteins. As molecular chaperones might be possible options,^38^, ^39^ the in situ gene editing to reverse the mutation is also promising, due to the rapid development of CRISPR/Cas9 DNA editing system. Given that both variants result in a G>A change (equivalent to C>T), it is probable that adenine base editors (ABEs) will be employed to facilitate the conversion of A•T to G•C base pairs in genomic DNA.^40^ Although our preliminary efforts to search for candidate short guide RNAs (sgRNAs) matching with ABEs have been limited in *HGSNAT*, due to the lack of suitable editing windows (data not shown), more evolved and improved versions of ABEs are expected to be employed in near future.

## AUTHOR CONTRIBUTIONS

**Hongjun Zhao:** Methodology (equal); resources (equal). **Lijing Wang:** Methodology (equal); resources (equal). **Mengfei Zhang:** Data curation (supporting). **Huakun Wang:** Data curation (supporting). **Sizhe Zhang:** Data curation (supporting). **Junjiao Wu:** Conceptualization (equal); supervision (equal); writing – original draft (equal); writing – review and editing (equal). **Yu Tang:** Conceptualization (equal); funding acquisition (lead); supervision (equal); writing – original draft (equal); writing – review and editing (equal).

## FUNDING INFORMATION

This study was funded by National Natural Sciences Foundation of China [No. 82271280 to YT and 82301433 to JJW], Hunan Provincial Natural Science Foundation of China [No. 2022JJ40824 to JJW], Scientific Research Project of Hunan Provincial Health Commission [No. B202303070054 to YT], Talents Startup Fund [No. 2209090550 to YT], Youth Science Fund [No. 2021Q04 to JJW] and Project Program of National Clinical Research Center for Geriatric Disorders [No. 2022LNJJ14 to HJZ] of Xiangya Hospital, Central South University, Changsha, China.

## CONFLICT OF INTEREST STATEMENT

The authors declare no conflict of interests.

## CONSENT FOR PUBLICATION

All participants have consented to publication.

## Supporting information


Data S1.

## Data Availability

Data are available from the corresponding author upon request.

## References

[1] Spahiu L , Behluli E , Peterlin B , et al. Mucopolysaccharidosis III: molecular basis and treatment. Pediatr Endocrinol Diabetes Metab. 2021;27(3):201‐208. 34743503 10.5114/pedm.2021.109270 PMC10228206

[2] Mouw JK , Ou G , Weaver VM . Extracellular matrix assembly: a multiscale deconstruction. Nat Rev Mol Cell Biol. 2014;15(12):771‐785. 25370693 10.1038/nrm3902 PMC4682873

[3] Paganini C , Costantini R , Superti‐Furga A , Rossi A . Bone and connective tissue disorders caused by defects in glycosaminoglycan biosynthesis: a panoramic view. FEBS J. 2019;286(15):3008‐3032. 31286677 10.1111/febs.14984

[4] Oussoren E , Brands MM , Ruijter GJ , der Ploeg AT , Reuser AJ . Bone, joint and tooth development in mucopolysaccharidoses: relevance to therapeutic options. Biochim Biophys Acta. 2011;1812(11):1542‐1556. 21827850 10.1016/j.bbadis.2011.07.013

[5] Valstar MJ , Ruijter GJ , van Diggelen OP , Poorthuis BJ , Wijburg FA . Sanfilippo syndrome: a mini‐review. J Inherit Metab Dis. 2008;31(2):240‐252. 18392742 10.1007/s10545-008-0838-5

[6] Beneto N , Vilageliu L , Grinberg D , Canals I . Sanfilippo syndrome: molecular basis, disease models and therapeutic approaches. Int J Mol Sci. 2020;21(21):7819. 33105639 10.3390/ijms21217819 PMC7659972

[7] Lloyd‐Evans E , Haslett LJ . The lysosomal storage disease continuum with ageing‐related neurodegenerative disease. Ageing Res Rev. 2016;32:104‐121. 27516378 10.1016/j.arr.2016.07.005

[8] Fedele AO . Sanfilippo syndrome: causes, consequences, and treatments. Appl Clin Genet. 2015;8:269‐281. 26648750 10.2147/TACG.S57672 PMC4664539

[9] Marco S , Pujol A , Roca C , et al. Progressive neurologic and somatic disease in a novel mouse model of human mucopolysaccharidosis type IIIC. Dis Model Mech. 2016;9(9):999‐1013. 27491071 10.1242/dmm.025171 PMC5047683

[10] Klein U , Kresse H , von Figura K . Sanfilippo syndrome type C: deficiency of acetyl‐CoA:alpha‐glucosaminide N‐acetyltransferase in skin fibroblasts. Proc Natl Acad Sci USA. 1978;75(10):5185‐5189. 33384 10.1073/pnas.75.10.5185 PMC336290

[11] Fan X , Zhang H , Zhang S , et al. Identification of the gene encoding the enzyme deficient in mucopolysaccharidosis IIIC (Sanfilippo disease type C). Am J Hum Genet. 2006;79(4):738‐744. 16960811 10.1086/508068 PMC1592569

[12] Hrebicek M , Mrazova L , Seyrantepe V , et al. Mutations in ™EM76* cause mucopolysaccharidosis IIIC (Sanfilippo C syndrome). Am J Hum Genet. 2006;79(5):807‐819. 17033958 10.1086/508294 PMC1698556

[13] Borges P , Pasqualim G , Giugliani R , Vairo F , Matte U . Estimated prevalence of mucopolysaccharidoses from population‐based exomes and genomes. Orphanet J Rare Dis. 2020;15(1):324. 33208168 10.1186/s13023-020-01608-0 PMC7672855

[14] Zelei T , Csetneki K , Voko Z , Siffel C . Epidemiology of Sanfilippo syndrome: results of a systematic literature review. Orphanet J Rare Dis. 2018;13(1):53. 29631636 10.1186/s13023-018-0796-4 PMC5891921

[15] Kong W , Meng Y , Zou L , Yang G , Wang J , Shi X . Mucopolysaccharidosis III in Mainland China: natural history, clinical and molecular characteristics of 34 patients. J Pediatr Endocrinol Metab. 2020;33(6):793‐802. 32447333 10.1515/jpem-2019-0505

[16] Li X , Xiao R , Chen B , et al. A novel mutation of SGSH and clinical features analysis of mucopolysaccharidosis type IIIA. Medicine (Baltimore). 2018;97(52):e13758. 30593151 10.1097/MD.0000000000013758 PMC6314651

[17] Li J , Xie H , Jiang Y . Mucopolysaccharidosis IIIB and mild skeletal anomalies: coexistence of NAGLU and CYP26B1 missense variations in the same patient in a Chinese family. BMC Med Genet. 2018;19(1):51. 29606097 10.1186/s12881-018-0562-4 PMC5880076

[18] Sambrook J , Fritsch E , Maniatis T . Molecular Cloning: A Laboratory Manual. 2nd ed. Cold Spring Harbor Laboratory Press; 1989.

[19] Frazer J , Notin P , Dias M , et al. Disease variant prediction with deep generative models of evolutionary data. Nature. 2021;599(7883):91‐95. 34707284 10.1038/s41586-021-04043-8

[20] Zhang HC , Xu SM , Fan X , Chung KW , Shen YF . Predicting functional effect of missense variants using graph attention neural networks. Nat Mach Intell. 2022;4:1017‐1028. 37484202 10.1038/s42256-022-00561-w PMC10361701

[21] Sundaram L , Gao H , Padigepati SR , et al. Predicting the clinical impact of human mutation with deep neural networks. Nat Genet. 2018;50(8):1161‐1170. 30038395 10.1038/s41588-018-0167-z PMC6237276

[22] Cheng J , Novati G , Pan J , et al. Accurate proteome‐wide missense variant effect prediction with AlphaMissense. Science. 2023;381(6664):eadg7492. 37733863 10.1126/science.adg7492

[23] Jiang TT , Fang L , Wang K . Deciphering "the language of nature": a transformer‐based language model for deleterious mutations in proteins. Innovation (Camb). 2023;4(5):100487. 37636282 10.1016/j.xinn.2023.100487 PMC10448337

[24] Danzi MC , Dohrn MF , Fazal S , et al. Deep structured learning for variant prioritization in Mendelian diseases. Nat Commun. 2023;14(1):4167. 37443090 10.1038/s41467-023-39306-7 PMC10345112

[25] Li Q , Wang K . InterVar: clinical interpretation of genetic variants by the 2015 ACMG‐AMP guidelines. Am J Hum Genet. 2017;100(2):267‐280. 28132688 10.1016/j.ajhg.2017.01.004 PMC5294755

[26] Hallgren J , Tsirigos DK , Pedersen MD , et al. Deep™HMM predicts alpha and beta transmembrane proteins using deep neural networks. bioRxiv. 2022;4(8):487609.

[27] Tsirigos KD , Peters C , Shu N , Kall L , Elofsson A . The TOPCONS web server for consensus prediction of membrane protein topology and signal peptides. Nucleic Acids Res. 2015;43(W1):W401‐W407. 25969446 10.1093/nar/gkv485 PMC4489233

[28] Crooks GE , Hon G , Chandonia JM , Brenner SE . WebLogo: a sequence logo generator. Genome Res. 2004;14(6):1188‐1190. 15173120 10.1101/gr.849004 PMC419797

[29] Jumper J , Evans R , Pritzel A , et al. Highly accurate protein structure prediction with AlphaFold. Nature. 2021;596(7873):583‐589. 34265844 10.1038/s41586-021-03819-2 PMC8371605

[30] Capriotti E , Fariselli P , Casadio R . I‐Mutant2.0: predicting stability changes upon mutation from the protein sequence or structure. Nucleic Acids Res. 2005;33:W306‐W310. 15980478 10.1093/nar/gki375 PMC1160136

[31] Li C , Chen Q , Wu J , et al. Identification and characterization of two novel noncoding tyrosinase (TYR) gene variants leading to oculocutaneous albinism type 1. J Biol Chem. 2022;298(5):101922. 35413289 10.1016/j.jbc.2022.101922 PMC9108984

[32] Prado‐Martinez J , Hernando‐Herraez I , Lorente‐Galdos B , et al. The genome sequencing of an albino Western lowland gorilla reveals inbreeding in the wild. BMC Genomics. 2013;14:363. 23721540 10.1186/1471-2164-14-363 PMC3673836

[33] Hessa T , Kim H , Bihlmaier K , et al. Recognition of transmembrane helices by the endoplasmic reticulum translocon. Nature. 2005;433(7024):377‐381. 15674282 10.1038/nature03216

[34] Seurig M , Ek M , von Heijne G , Fluman N . Dynamic membrane topology in an unassembled membrane protein. Nat Chem Biol. 2019;15(10):945‐948. 31501590 10.1038/s41589-019-0356-9

[35] Patrick AE , Karamyshev AL , Millen L , Thomas PJ . Alteration of CFTR transmembrane span integration by disease‐causing mutations. Mol Biol Cell. 2011;22(23):4461‐4471. 21998193 10.1091/mbc.E11-05-0396 PMC3226467

[36] Xu L , Xia M , Guo J , et al. Impairment on the lateral mobility induced by structural changes underlies the functional deficiency of the lupus‐associated polymorphism FcgammaRIIB‐T232. J Exp Med. 2016;213(12):2707‐2727. 27799621 10.1084/jem.20160528 PMC5110019

[37] Hu W , Zhang Y , Sun X , et al. FcgammaRIIB‐I232T polymorphic change allosterically suppresses ligand binding. eLife. 2019;8:8. 10.7554/eLife.46689 PMC6711707 31343409

[38] Feldhammer M , Durand S , Pshezhetsky AV . Protein misfolding as an underlying molecular defect in mucopolysaccharidosis III type C. PLoS One. 2009;4(10):e7434. 19823584 10.1371/journal.pone.0007434 PMC2757673

[39] Matos L , Canals I , Dridi L , et al. Therapeutic strategies based on modified U1 snRNAs and chaperones for Sanfilippo C splicing mutations. Orphanet J Rare Dis. 2014;9:180. 25491247 10.1186/s13023-014-0180-y PMC4279800

[40] Gaudelli NM , Komor AC , Rees HA , et al. Programmable base editing of A*T to G*C in genomic DNA without DNA cleavage. Nature. 2017;551(7681):464‐471. 29160308 10.1038/nature24644 PMC5726555

