# Supplementary material for: Identification and characterization of novel genetic variants in the first Chinese family of mucopolysaccharidosis IIIC (Sanfilippo C syndrome)
Source: J Cell Mol Med. 2024 Apr 13;28(8):e18307. doi: 10.1111/jcmm.18307 (PMC11015392; doi:10.1111/jcmm.18307)
Supplement: Supplementary file 1 — Data S1. [file JCMM-28-e18307-s001.docx]

## Supplementary Figures


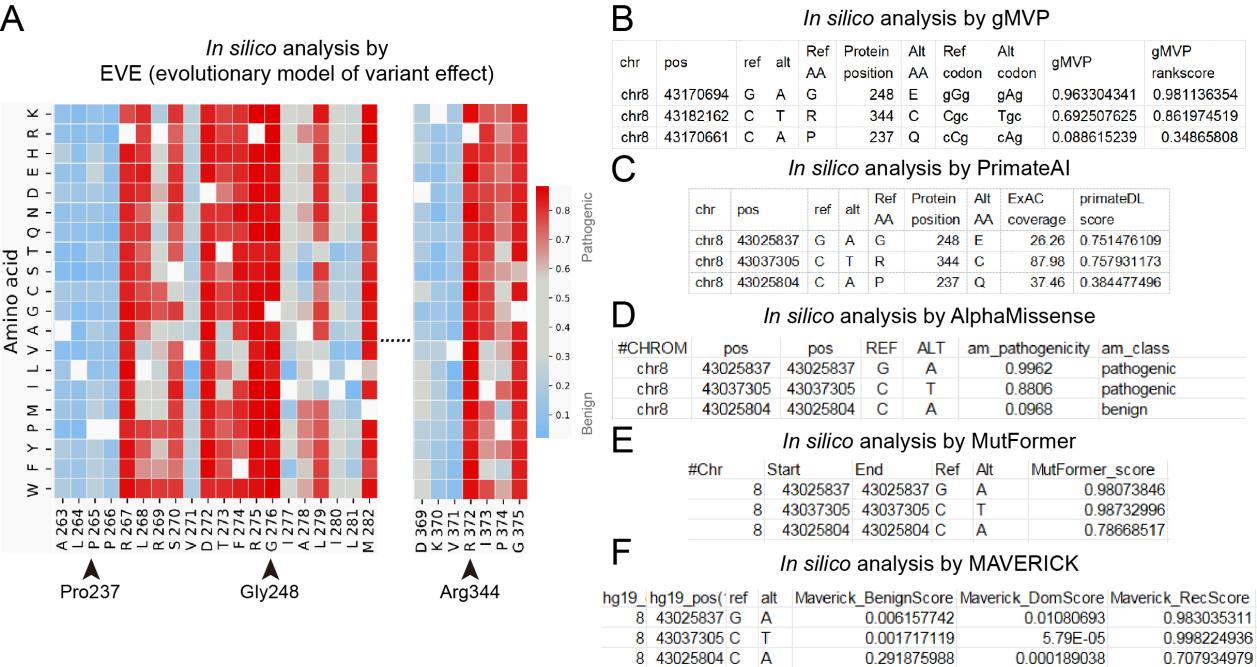


**Supplementary Fig. S1. Further *in silico* analysis of pathogenicity.** The pathogenicity was further assessed with the most updated algorithms, empowered by machine/deep learning or neural network model, such as EVE (**A**), gMVP (**B**), PrimateAI (**C**), AlphaMissense (**D**), MutFormer (**E**) and MAVERICK (**F**). The p.Gly248Glu/p.Arg344Cys variants were all recognized as highly pathogenic.

## Supplementary Tables

**Supplementary Table S1. *In silico* analysis of HGSNAT protein stability.** The effect of protein mutations of HGSNAT (UniProt Q68CP4) protein stability was evaluated by I-Mutant 2.0, and showed that both p.Gly248Glu/p.Arg344Cys variants may lead to large decrease of stability.

| **Position** | **WT** | **NEW** | **pH** | **T** | **SVM2 Prediction Effect** | **RI** | **DDG Value Prediction** |
| --- | --- | --- | --- | --- | --- | --- | --- |
| 248 | Gly | Glu | 7.0 | 25 | Decrease | 2 | -0.76 kcal/mol |
| 344 | Arg | Cys | 7.0 | 25 | Decrease | 7 | -1.00 kcal/mol |

Footnote:

RI: Reliability Index; WT: Amino acid in Wild-Type Protein; NEW: New Amino acid after Mutation; T: Temperature in Celsius unit; pH: -log[H+]

Stability Predictors:

DDG Value:

DG(New Protein)-DG(Wild Type) in kcal/mol

Binary Classification (SVM2):

DDG<0: Decrease Stability

DDG>0: Increase Stability

Ternary Classification (SVM3):

DDG<-0.5: Large Decrease of Stability

DDG>0.5: Large Increase of Stability

-0.5<=DDG<=0.5: Neutral Stability
